# Effect of IL-10 Deficiency on TGFβ Expression during Fatal Alphavirus Encephalomyelitis in C57Bl/6 Mice

**DOI:** 10.3390/v14081791

**Published:** 2022-08-16

**Authors:** Nina M. Martin, Diane E. Griffin

**Affiliations:** W. Harry Feinstone Department of Molecular Microbiology and Immunology, Johns Hopkins Bloomberg School of Public Health, Baltimore, MD 21205, USA

**Keywords:** neuroadapted Sindbis virus, innate lymphoid cells, inflammation, transforming growth factor beta, encephalomyelitis, mice

## Abstract

Sindbis virus (SINV) causes viral encephalitis in mice with strain-dependent virulence. Fatal encephalomyelitis in C57Bl/6 mice infected with a neuroadapted strain of SINV (NSV) is an immunopathogenic process that involves Th17 cells modulated by the regulatory cytokine IL-10. To further characterize the pathogenic immune response to NSV, we analyzed the regulation of transforming growth factor (TGF)-b in both wild-type (WT) and IL-10-deficient mice. NSV infection upregulated the expression of TGFb1 and TGFb3 in the central nervous system (CNS). In the absence of IL-10, levels of brain *Tgfb1* mRNA and brain and spinal cord mature active TGFβ1 and TGFβ3 proteins were higher than in WT mice. Compared to WT mice, IL-10-deficient mice had more TGFβ1-expressing type 3 innate lymphoid cells (ILC3s) and CD4^+^ T cells infiltrating the CNS, but similar numbers in the cervical lymph nodes. Expression of glycoprotein A repetitions predominant protein (GARP) that binds pro-TGFb on the surface of regulatory T cells was decreased on CNS cells from IL-10-deficient mice. Higher CNS TGFb was accompanied by more expression of TGFbRII receptor, activation of SMAD transcription factors, increased *PCKα* mRNA, and more RORγt-positive and IL-17A-expressing cells. These results suggest a compensatory role for TGFβ in the absence of IL-10 that fosters Th17-related immunopathology and more rapid death after NSV infection.

## 1. Introduction

Viral encephalitis is life-threatening and an important cause of long-term disability worldwide, yet the mechanisms of neurocognitive damage remain poorly understood, making the development of therapeutic interventions difficult [1,2]. Arthropod-borne encephalitic viruses are important causes of seasonal disease in the Americas and include the alphaviruses eastern equine encephalitis [EEE] and Venezuelan equine encephalitis [VEE] viruses [3]. EEE has an estimated 35% mortality, and almost all survivors are left with neurological sequelae [4]. A recent outbreak of VEEV in Colombia and Venezuela resulted in an estimated 75,000 to 100,000 cases [5,6]. As the emergence and spread of these neurotropic viruses increase, the need to understand the mechanisms responsible for severe and fatal disease becomes more critical.

Sindbis virus (SINV) is the prototypic alphavirus, and although disease in humans is usually associated with rash and arthritis [7], the disease in mice parallels viral-induced encephalomyelitis with neurons as the primary target cells [8,9]. As with EEEV and VEEV, SINV outcomes are dependent on the age of the host and the virulence of the virus strain [6,10,11,12,13,14,15,16]. A neuroadapted strain of SINV (NSV) causes lethal encephalitis in 4–6 week old C57Bl/6 mice, while mice recover from infection with less virulent strains [8,12]. Previous studies have shown that fatal NSV-induced encephalomyelitis is an immunopathologic process driven by CD4^+^ T cells, although knowledge of the mechanisms of immune-mediated neuronal damage remains incomplete [17,18,19,20].

Because T cells are implicated in immunopathogenesis, there is a need to understand how the cellular immune response and inflammation are regulated during viral infection of the central nervous system (CNS). Important local regulators are the cytokines interleukin (IL)-10 and transforming growth factor (TGF)-b [21]. Previous studies have shown that the production of IL-10, a cytokine that controls inflammation in part by regulating differentiation of CD4^+^ T cells, is an important determinant of the outcome of SINV-induced encephalomyelitis [22,23,24,25]. IL-10 suppresses the development of Th1 and Th17 cells that can be mediators of immune pathology [26,27,28], and mice deficient in IL-10 develop accelerated fatal encephalomyelitis after NSV infection associated with an early increase in pathogenic Th17 cells, neutrophils and *Il17a* mRNA [22]. In the current studies, we have further characterized immune responses in the CNS during NSV infection in both WT and IL-10-deficient mice to identify potential contributions of TGF-β to the pathogenic Th17 response.

TGF-b isoforms 1, 2 and 3 are members of the TGF-b subfamily in a large superfamily of regulatory proteins that includes bone morphogenetic proteins, growth and differentiation factors, and activins [29,30]. These highly regulated proteins are synthesized as precursor proteins that must be proteolytically processed to produce the active proteins, so regulation is primarily after transcription. Bioavailability of the processed active protein is further controlled by continued interaction with the pro-domain and binding to locally produced soluble, matrix and membrane proteins [30,31,32]. TGFb is produced by both immune and non-immune cells, including neural cells, and regulates the initiation, development, and resolution of immune responses by binding to heterodimeric receptors and activating SMAD family transcription factors as well as signaling through SMAD-independent pathways [31,33].

To determine the role of TGFβ in the response to NSV infection, we have analyzed the immune response in the CNS of IL-10-deficient mice in comparison to WT mice and show that accelerated morbidity and mortality are associated with greater TGFb pathway activation and Th17-related responses.

## 2. Materials and Methods

### 2.1. Virus Infection of Mice and Tissue Collection

The NSV, TE12 and TE strains of SINV [12] were grown and assayed by plaque formation in BHK-21 cells. C57BL/6J (B6 WT) and B6.129P2-Il10^tm1Cgn/J^ (B6 IL10-/-) mice were purchased from Jackson Laboratories and bred in-house. Next, 4- to 6-week-old sex and age-matched mice were infected intranasally with 10^5^ plaque-forming units (pfu) NSV diluted in PBS. Disease severity was assessed using a previously described scoring system [22]. Briefly, 0 = no signs of disease; 1 = abnormal hind limb and tail posture, ruffled fur, and/or hunched back; 2 = one hind-limb paralyzed; 3 = both hind-limbs or full-body paralysis; 4 = death.

For the collection of cervical lymph node (CLN), brain and spinal cord tissues, mice were anesthetized with isoflurane and perfused with ice-cold PBS. Tissues were used fresh or frozen at −80 °C. Subsequently, 10% weight per volume tissue homogenates were made in PBS and clarified by centrifugation. All experiments were performed according to protocols approved by the Johns Hopkins University Animal Care and Use Committee.

### 2.2. mRNA and Protein Analyses

RNA was isolated from frozen brain and spinal cord tissues with the RNeasy Lipid Mini RNA Isolation Kit (Qiagen, Hilden, Germany). A nanodrop spectrophotometer was used to quantify RNA and cDNA prepared with the High Capacity cDNA Reverse Transcription Kit (Life Technologies, Carlsbad, CA, USA) using 0.5–2.5 μg RNA. Quantitative real-time PCR was performed using 2.5 μL cDNA, TaqMan gene expression arrays (*pkca*, *tgfb1*, *tgfb3*, *smad2* and *smad3*), and 2× Universal PCR Mastermix (Applied Biosystems, Waltham, MA, USA). *Gapdh* mRNA was quantified using the rodent primer and probe set (Applied Biosystems). Reactions were run with the following conditions on an Applied Biosystems 7500 real-time PCR machine: 50 °C for 2 min, 95 °C for 10 min, 95 °C for 15 s, and 60 °C for 1 min for 50 cycles. The target gene Ct value was normalized to the Ct value of *Gapdh* to determine the transcript level. The normalized value was used to calculate gene expression relative to the average of the uninfected WT control (ΔΔCt method).

Levels of active TGFβ1 and TGFβ3 proteins in the brain and spinal cord homogenates were measured by enzyme immunoassay (EIA) according to the manufacturer’s instructions (R & D Systems, Minneapolis, MN, USA).

### 2.3. Mononuclear Cell Isolation

Mononuclear cells were isolated from fresh CLNs and brains collected from WT or IL10-/- mice after NSV infection. CLNs were pooled in 10 mL of RPMI/1% FBS and homogenized in C tubes using the GentleMACS spleen program 1 for 2 cycles (Miltenyi, Bergisch Gladbach, Germany). Two brains collected in ice-cold HBSS were pooled per C tube containing 4 mL of enzyme digest mix (RPMI, 1% FBS, 1 mg/mL collagenase (Roche, Basel, Switzerland), 0.1 mg/mL DNase (Roche)) and dissociated with scissors. Further dissociation was performed using the GentleMACS brain program 3 (5× with two 15 min 37 °C incubations and gentle rocking). Suspensions were filtered through 70 mm strainers and centrifuged. Cells were treated with red blood cell lysis buffer (Sigma, St. Louis, MO, USA) for 3 min and then washed with PBS. Cell pellets were resuspended in 30% percoll, underlaid with 70% percoll and centrifuged at 4 °C. for 30 min at 850× *g*. The debris layer was removed, and mononuclear cells were collected from the interface, washed, and suspended in PBS containing 2 mM EDTA. Live cells were counted using trypan blue exclusion.

### 2.4. Flow Cytometry

Approximately 10^6^ cells in PBS with 2 mM EDTA were stained with the violet Live/Dead Fixable Cell Stain Kit (Invitrogen, Waltham, MA, USA), blocked with rat anti-mouse CD16/CD32 (BD Pharmingen, San Diego, CA, USA), diluted in PBS with 2 mM EDTA and 0.5% BSA, surface-stained for 30 min on ice, fixed and resuspended in FACS buffer. Conjugated antibodies (BD Pharmingen or eBioscience, San Diego, CA, USA) to the following proteins were used: CD45-FITC, CD3-APC, CD4-PerCP-Cy5.5, GARP-PE, lineage-FITC, and IL-7Ra-PE Cy7. Cell type definitions were: T cells (CD3^+^), CD4 T cells (CD3^+^CD4^+^), CD8 T cells (CD3^+^CD8^+^) and ILCs (Lin^-^IL-7Ra^+^).

For intracellular staining to detect cytokines and activated transcription factors, 2–3 × 10^6^ cells in RPMI containing 1% FBS were stimulated with phorbol-12-myristate 13-acetate (PMA; 50 ng/mL) and ionomycin (1 μg/mL) in the presence of GolgiPlug-brefeldin A (BD Pharmingen) for 4 h. After surface staining, cells were fixed and permeabilized using CytoFix/CytoPerm (BD Pharmingen) and stained for 30 min on ice with anti-TGFβ1-PE, anti-TGFβ3-biotin, streptavidin-PE or anti-p-SMAD2/3 (BD Pharmingen or eBioscience) and resuspended in 500 μL FACS buffer. Assessment of RORγt expression used the Foxp3 Buffer Set (eBiosciences). After surface staining, cells were fixed, permeabilized and stained for RORγt to identify ILC3 cells (Lin^-^IL-7Ra^+^ RORγt^+^). Data were acquired with a BD FACS Canto II flow cytometer using FACS Diva software (version 6.0) and analyzed using FlowJo version 10.3.0. The ILC3 gating strategy is shown in Supplementary Figure S1.

### 2.5. Statistical Analysis

Data from two to four independent experiments with 6–10 mice per group were used. Differences between groups over time were determined using two-way ANOVA and Bonferroni posttests. Differences between groups at a single time point were determined using an unpaired, two-tailed Student’s *t*-test with a 95% confidence interval. All statistical analyses were calculated in GraphPad Prism 5 (v5.01).

## 3. Results

### 3.1. CNS Expression of TGFb after SINV Infection

**Figure 1:**
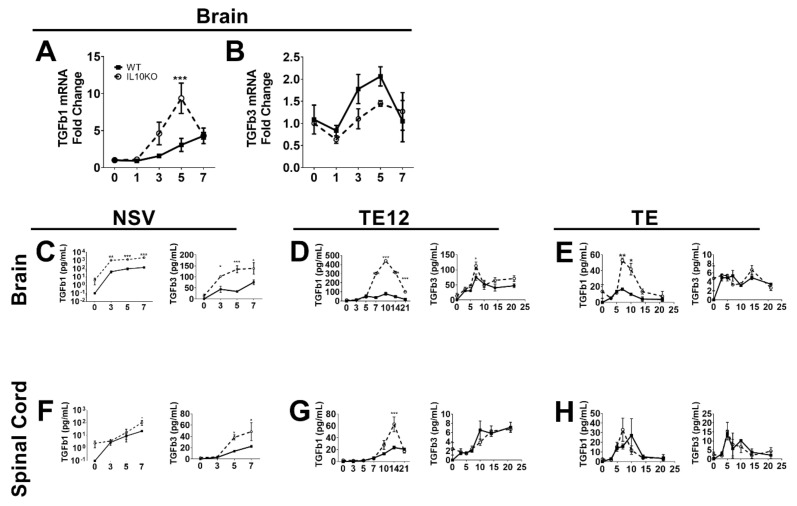
Induction of TGFb in response to CNS infection with SINV strains of differing virulence. Analysis of TGFβ1 (**A**) and TGFβ3 (**B**) mRNAs in the brains of WT (filled square, solid line) and IL-10-/- (open circle, dashed line) mice during NSV infection. Gene Ct values were normalized to *Gapdh*, and fold change was calculated relative to uninfected WT controls (ΔΔCt). Data are pooled from two independent experiments and presented as the mean ± SEM from 6 mice at each time point. *** *p* < 0.001. Levels of active TGFβ1 and TGFβ3 were measured in homogenates of brain (**C**–**E**) and spinal cord (**F**–**H**) tissues from mice infected with NSV (**C**,**F**), TE12 (**D**,**G**) or TE (**E**,**H**) by EIA. Data were pooled from 2 independent experiments and represent the means ± SEM for 8 mice at each time point; * *p* < 0.05; ** *p* < 0.01; *** *p* < 0.001.

The regulatory cytokine TGFb has overlapping suppressive functions with IL-10 and is of particular importance for regulating inflammation in the CNS [34,35,36]. Previous studies have shown that IL-10 is produced in the CNS in response to infection with NSV and that immunopathology and fatal paralysis are accelerated in the absence of IL-10 [22]. To determine whether TGFb mRNAs are induced in response to CNS infection with NSV and whether IL-10 deficiency alters this expression, we performed RT-qPCR on RNA isolated from brains of WT and IL10-/- B6 mice infected intranasally with NSV (Figure 1A,B). Infection increased brain expression of both *Tgfb1* and *Tgfb3* mRNAs beginning at 3 days and a maximal 5–7 days after infection. Levels of *Tgfb1* mRNA were higher in IL-10-/- mice than WT mice (Figure 1A), but no differences were identified in levels of *Tgfb3* mRNA (Figure 1B).

Because levels of biologically active TGFβ protein are determined post-transcriptionally [37], we measured mature TGFβ1 and TGFβ3 proteins by EIA. Brain and spinal cord tissues from NSV-infected mice had increased levels of activated TGFβ1 and TGFβ3 proteins, and IL10-/- mice had higher levels than WT mice. Brain levels increased within 3 days (Figure 1C), and spinal cord levels increased by 5 days after infection (Figure 1F), perhaps reflecting the progressive spread of the virus from the brain to the spinal cord after intranasal infection [38,39]. Therefore, IL-10 deficiency results in greater production of active TGFβ in the CNS after NSV infection than in WT mice. To determine if these changes were unique to NSV, we also measured CNS levels of active TGFb1 and TGFb3 after infection with moderately virulent TE12 (Figure 1D,G) and relatively avirulent TE (Figure 1E,H) recombinant strains of SINV. Compared to NSV infection, WT mice showed little change, but levels of TGFb1 increased in IL-10-deficient mice.

### 3.2. Cellular Sources of Increased TGFβ

**Figure 2:**
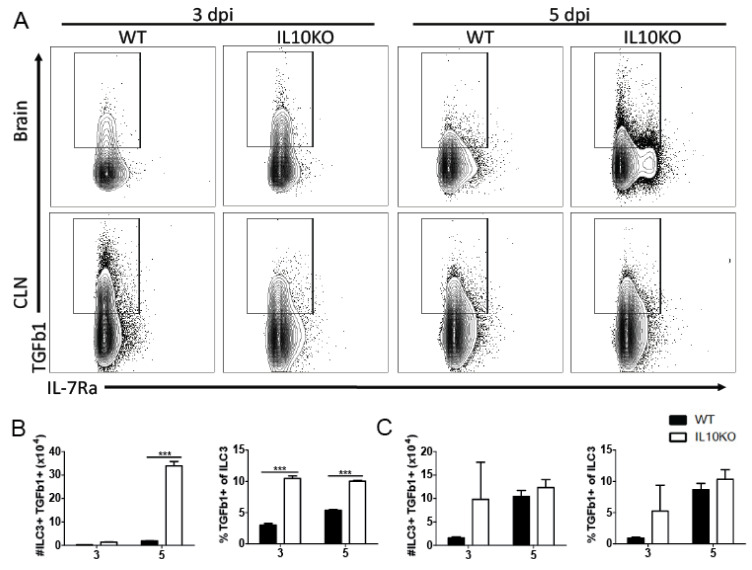
TGFβ production by ILC3s. Intracellular cytokine staining and flow cytometry were performed on cells isolated from the brains and CLNs from WT (black bar) or IL10-deficient (KO, white bar) mice 3 and 5 days after NSV infection. (**A**) Representative flow cytometry plots. ILC3s were identified as Lineage-IL-7Ra^+^RORγt^+^ cells. Percent and number of ILC3s producing TGFβ1 were measured in the brain (**B**) and CLN (**C**). Bars represent mean ± SEM from 10 mice per group. *** *p* < 0.001.

**Figure 3:**
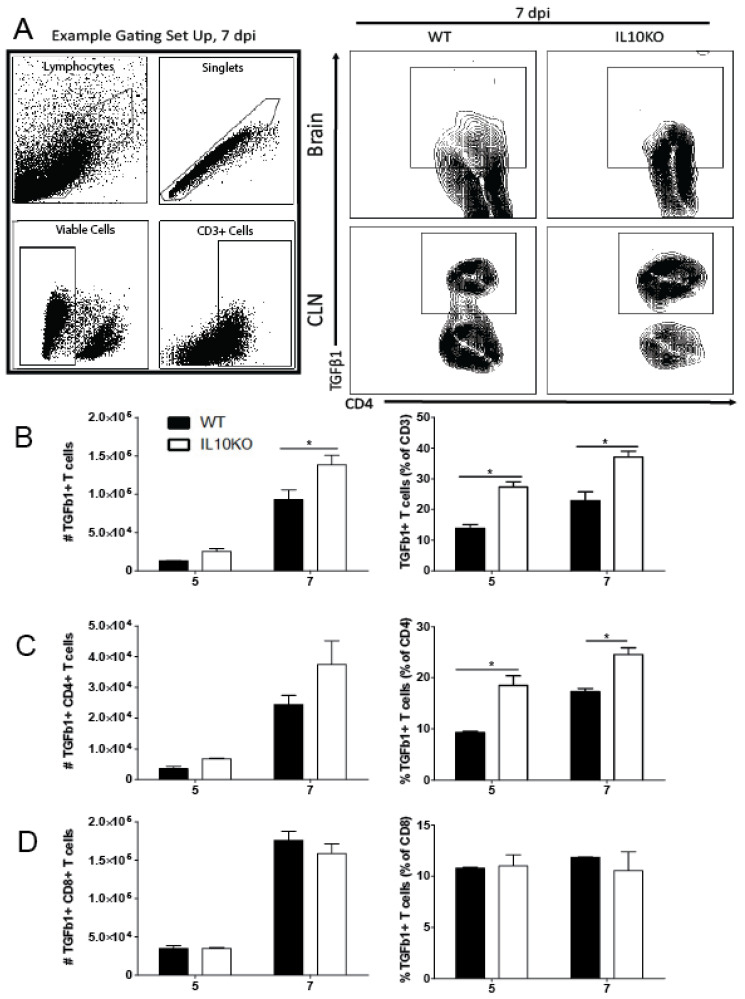
TGFβ production by T cells. Cells were isolated from brains and CLNs of WT (black bars) or IL10-/- (KO, white bars) mice 5 and 7 days after NSV infection. (**A**) Representative flow cytometry plots and gating strategy for brain T cells from 7 days after infection. Total CD3^+^ T cell numbers were not different. (**B**) CD3^+^ T cells producing TGFβ1 in brains were measured as percent and number of live cells. (**C**) CD4^+^ T cells were measured in brains as a percent and number of CD3^+^ T cells. (**D**) CD8^+^ T cells were measured in brains as percent and number of CD3^+^ T cells. Bars represent mean ± SEM from 10 mice per group. * *p* < 0.05.

Lymphocytes are important sources of TGFb, with TGFb1 most abundantly produced [31]. Because high levels of TGFβ proteins were present in the brain by 3 days after infection and continued to increase coincident with the infiltration of inflammatory cells into the CNS, we used flow cytometry to assess TGFb1 production by ILCs that infiltrate the brain early after infection [25] (Figure 2) as well as by CD4^+^ and CD8^+^ T cells, which accumulate later (Figure 3). Cells isolated 3 and 5 days after infection from the CLNs, where the immune response is induced, and from brains identified as ILC3s (lineage-IL7Ra^+^RORgt^+^, Supplementary Figure S1) were assessed for production of TGFβ1 by intracellular cytokine staining (Figure 2A). There were no differences in percent or number of TGFβ1-positive type 3 ILCs in CLN on day 3 or 5 after infection (Figure 2C). However, the brains of IL-10-/- mice had a higher percentage of ILC3 cells producing TGFβ1 3 days after infection, and by day 5, both the number and percentage of TGFβ1-positive ILC3s were higher in IL-10-/- mice than WT mice (Figure 3B). Therefore, ILC3s were an early source of TGFβ1 in the CNS after NSV infection that was increased in IL-10-deficient mice.

Previous studies showed that IL-10 deficiency does not impact the recruitment of CD4^+^ or CD8^+^ T cells into the CNS in response to NSV infection but does alter T cell differentiation and production of cytokines, resulting in more pathogenic Th17 cells [22]. To determine whether IL-10 deficiency affected the numbers or percentages of T cells producing TGFβ1, cells from the brains of WT and IL10-/- mice were examined by intracellular cytokine staining and flow cytometry (Figure 3). IL-10-deficient mice had higher numbers on day 5 and higher numbers and percentages on day 7 of CD3^+^ T cells producing TGFβ1 (Figure 3B). Of the CD3^+^ cells, a higher percentage of CD4^+^ T cells (Figure 3C), but not CD8^+^ T cells (Figure 3D), from IL-10-deficient mice produced TGFβ1 at both 5 and 7 days after infection compared to WT mice. Therefore, in IL-10-/- mice, CD4^+^ T cells were a continued source of increased TGFb later in the CNS inflammatory process.

### 3.3. Changes in Lymphocyte Expression of Surface Proteins That Bind TGFβ

**Figure 4:**
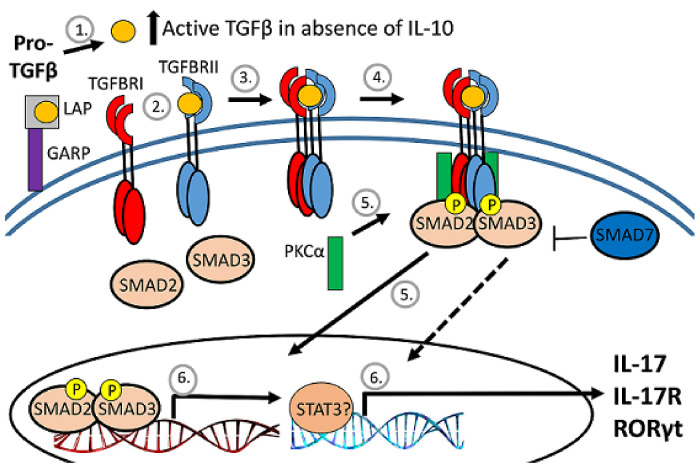
Diagram of TGFb regulation. After cleavage of proTGFb, TGFb remains associated with the latency-associated protein (LAP) pro-domain and inactive. The LAP/TGFb complex can be tethered to the surface of regulatory T cells by association with GARP. When released from LAP, TGFb binds to the heterodimeric TGFb receptor to activate serine/threonine kinases to phosphorylate and activate SMAD transcription factors that translocate to the nucleus.

TGFβ biologic activity is tightly regulated, beginning with production as a pro-form that is cleaved intracellularly by a furin-like protease into active TGFβ and the latency-associated protein (LAP) pro-domain (Figure 4). LAP remains noncovalently associated with TGFβ and inhibits receptor interaction. The release of TGFβ from the LAP complex for interaction with the TGFb receptor is accomplished primarily by the interaction of the RGD motif on LAP with integrin [32,40]. Prior to TGFb release, the LAP-TGFβ complex either interacts with the latent TGFβ-binding protein (LTBP) for retention by the extracellular matrix or with glycoprotein A repetitions predominant protein (GARP) on activated FoxP3-positive Tregs to become tethered to the cell surface [32,37,41,42].

**Figure 5:**
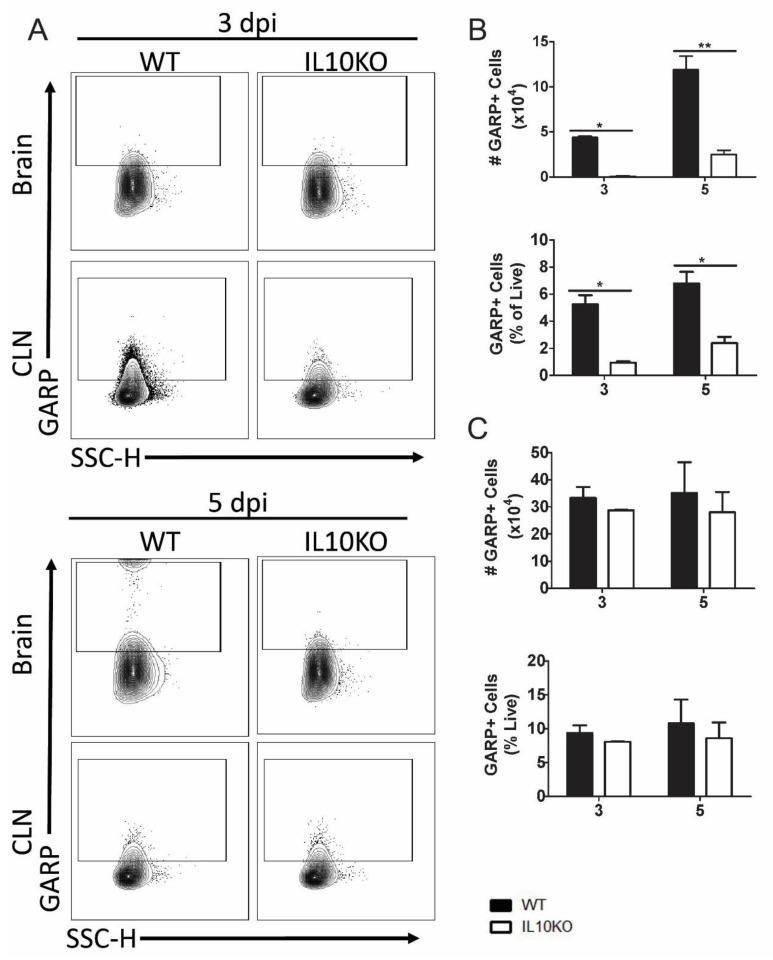
Cellular expression of GARP. Flow cytometric analysis of isolated cells pooled from the brains (n = 10) and CLNs of WT (black bars) or IL10-/- (white bars, KO) mice at 3 and 5 days after infection. (**A**) Representative flow cytometry plots. Total GARP^+^ cells were measured as percentage and number in brains (**B**) and CLNs (**C**). The data represent the mean ± SEM from three independent experiments. * *p* <0.05, ** *p* < 0.01.

The expression of GARP is a marker for activated Tregs, and GARP^+^CD4^+^CD25^+^ cells are increased in blood during chronic lentivirus infections [43,44]. To determine whether the development of GARP^+^CD4^+^ T cells in response to NSV infection was affected by IL-10 deficiency, GARP expression on cells isolated from brains and CLNs was examined by flow cytometry (Figure 5A). Lymphocytes isolated from the brains (Figure 5B), but not the CLNs (Figure 5C), of IL10-/- mice had lower numbers and percentages of GARP^+^ cells 3 and 5 days after infection than WT mice.

**Figure 6:**
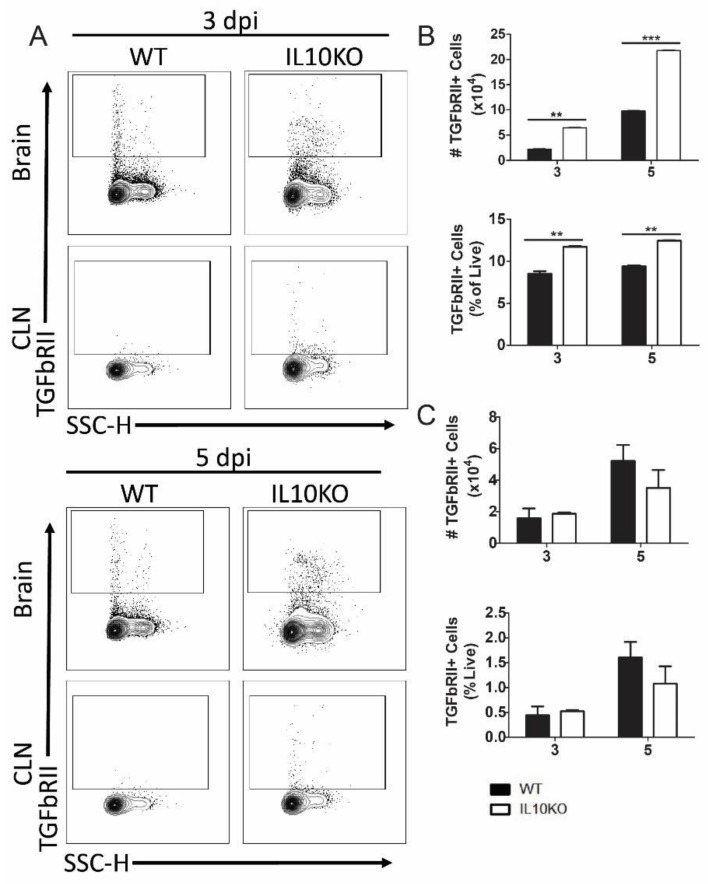
Cellular expression of TGFβ receptor II (TGFbRII). Flow cytometric analysis of isolated cells pooled from the brains (n = 10) and CLNs of WT (black bars) and IL10-/- (white bars, KO) mice at 3 and 5 days after NSV infection. (**A**) Representative flow cytometry plots. Total TGFbRII^+^ cells were measured as percentage and number in brains (**B**) and CLN (**C**). The data represent the mean ± SEM from three independent experiments; ** *p* < 0.05, *** *p* < 0.001.

To determine the impact of IL-10 deficiency on TGFβ receptor expression, lymphocytes isolated from brains and CLNs of WT or IL10-/- mice were assessed for TGFβRII expression (Figure 6A). Cerebral lymphocytes from IL-10-/- mice had higher numbers and percentages of cells expressing TGFβRII compared to cells from WT mice at both 3 and 5 days after infection (Figure 6B). There were no significant differences in the number or percent of TGFβRII-positive cells in the CLNs (Figure 6C).

### 3.4. Effect of IL-10 Deficiency on Expression and Activation of Smad Transcription Factors

**Figure 7:**
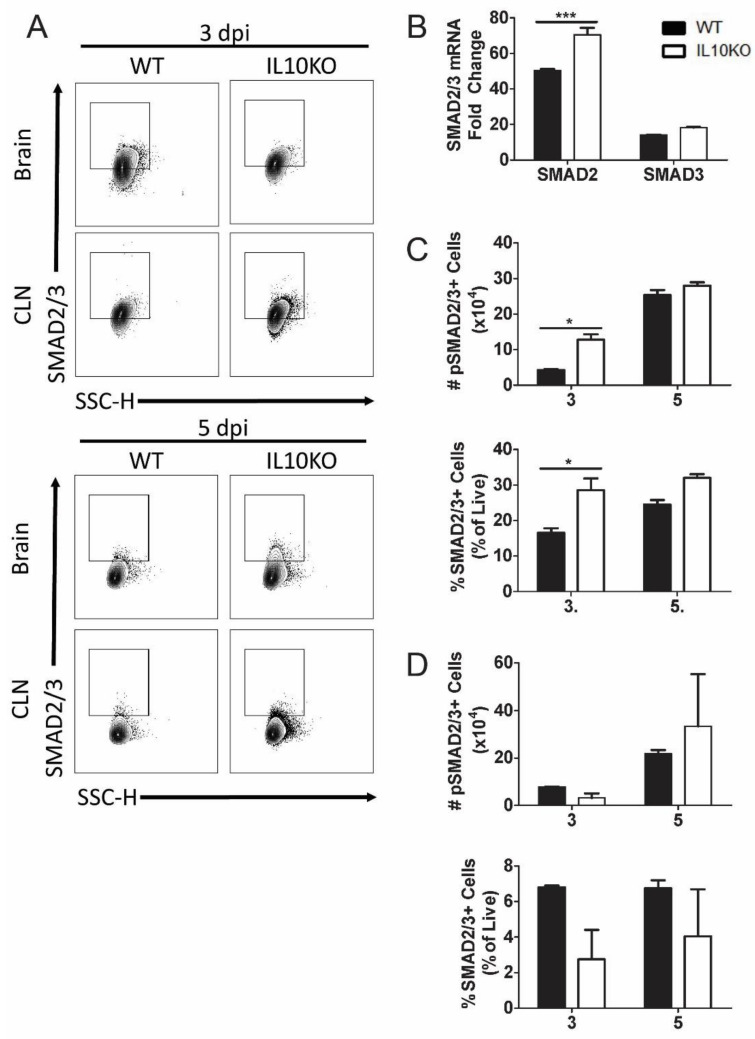
Expression and activation of SMAD2/3. Analysis of expression of SMAD2 and SMAD3 mRNAs in brains and phosphorylated SMAD2/3 in cells isolated from the brains and CLNs of WT and IL-10-deficient NSV-infected mice. (**A**) Representative flow cytometry plots. (**B**) Analysis of SMAD2 and SMAD3 mRNAs in the brains of WT (black bar) and IL-10-/- (white bar) mice on day 7 after infection. Gene Ct values were normalized to *Gapdh*, and fold change was calculated relative to uninfected WT controls (ΔΔCt). Data are pooled from two independent experiments and presented as the mean ± SEM from 6 mice in each group; *** *p* < 0.001. (**C**,**D**) Number and percentage of live cells positive for SMAD2/3 (lower panels) and phospho-SMAD2/3 (upper panels) in brains (**C**) and CLNs (**D**). Experiment repeated 3×. Data are presented as the mean ± SEM; * *p* < 0.05.

TGFβ binding to its cognate receptors, TGFβRI and TGFβRII, activates a signal transduction pathway that results in phosphorylation and activation of transcription factors SMAD2 and SMAD3 as well as Smad-independent signaling pathways (Figure 4) [40]. Phospho-SMAD2/3 regulates cytokine responses primarily by controlling the expression of transcription factors necessary for CD4^+^ T cell differentiation. TGFβ inhibits the differentiation of Th1 and Th2 cells by blocking the expression of *Tbet* and *Gata3* and promoting differentiation of Th17 and Tregs through dose-dependent induction of *RORγt* and *FoxP3* [33,40,45,46]. To assess SMAD2 and SMAD3 expression, mRNA was measured by RT-qPCR at the peak of CNS inflammation 7 days after infection (Figure 7B). *Smad2* mRNA expression was higher in the brains of IL10-/- mice compared to WT mice. To assess SMAD2/3 activation, phosphorylated SMAD2/3 expression in CLN and brain lymphocytes was analyzed 3 and 5 days after NSV infection (Figure 7A). Greater numbers and percentages of cells from the brains of IL10-/- mice were positive for phospho-SMAD2/3 on day 3 after NSV infection compared to WT mice (Figure 7C). There were no significant differences in percentages or numbers of phospho-SMAD2/3-positive cells isolated from CLN (Figure 7D).

### 3.5. IL10 Deficiency and TGFβ Upregulation Associated with Expression of Pkca and RORgt

**Figure 8:**
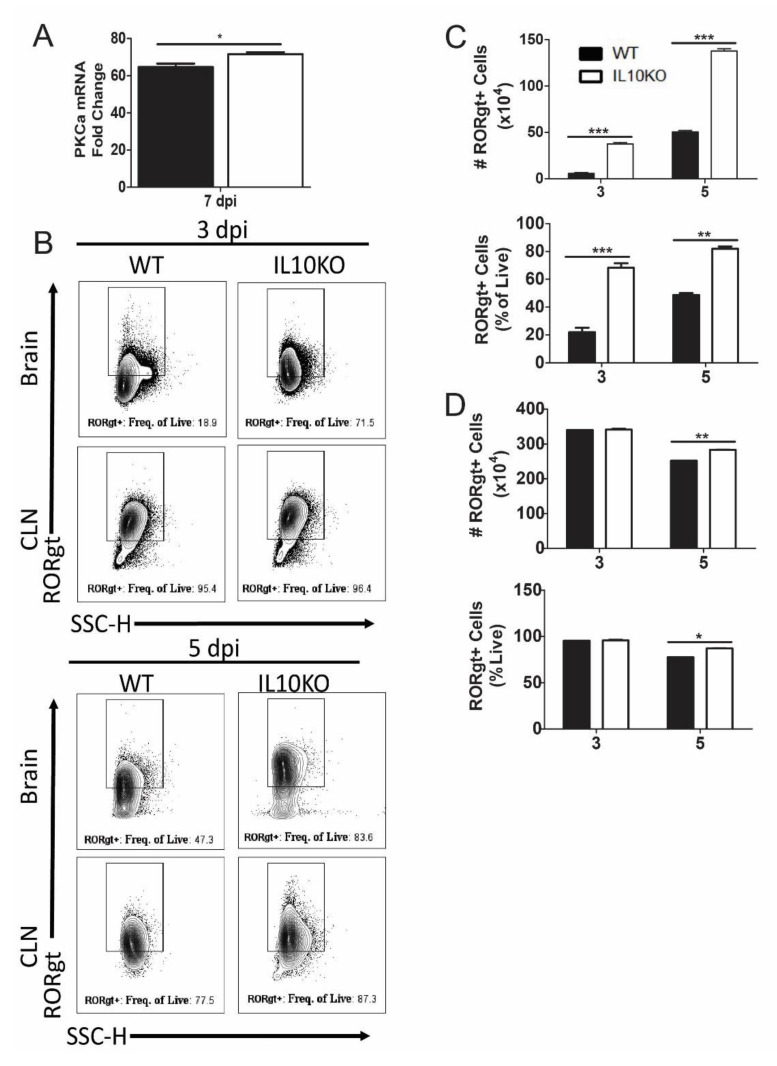
Expression of transcription factors PKCa and RORgt. (**A**) Analysis of *Pkcα* mRNA in the brains of WT (black bar) and IL-10-deficient (white bar, KO) mice 7 days after NSV infection. Gene Ct values were normalized to *Gapdh,* and fold change was calculated relative to uninfected controls (ΔΔCt). Data are pooled from two independent experiments and presented as the mean ± SEM from 6 mice in each group; * *p* < 0.05. (**B**–**D**) Flow cytometric analysis of cells expressing RORγt in WT (black bars) and IL10-/- (white bars) mice. (**B**) Representative flow cytometry plots. Number and percent of live cells in brains (**C**) and CLNs (**D**) expressing RORgt. Data are pooled from two independent experiments and presented as the mean ± SEM from 10 mice per time point; * *p* < 0.05, ** *p* < 0.01, *** *p* < 0.001.

Differentiation of IL-17A-producing T cells requires expression of PKCα, RORγt, and STAT3, which can be induced through SMAD-independent as well as SMAD-dependent TGFβ signaling pathways [45,47,48]. For example, PKCα-deficient cells demonstrated a defect in SMAD-dependent IL-2 suppression, STAT3 DNA binding within the *Il17a* promoter, and lack of IL-17A production [48]. Analysis of *PKCα* mRNAs in the brains showed increased levels in IL10-/- mice relative to WT mice (Figure 8A). Because RORγt regulation of IL-17 is common to many cell types, including T cells, neutrophils, and ILCs, RORγt expression was measured in total isolated lymphocytes from the brain and CLN (Figure 8B). Numbers and percentages of lymphocytes positive for RORγt were higher in the brains of IL10-/- mice compared to WT mice (Figure 8C), and in CLN, there were significantly higher percents and numbers of RORγt^+^ cells on day five, but not day three, after infection (Figure 8D).

### 3.6. IL10 Deficiency and TGFβ Upregulation Associated with IL-17A-Producing ILC3s

**Figure 9:**
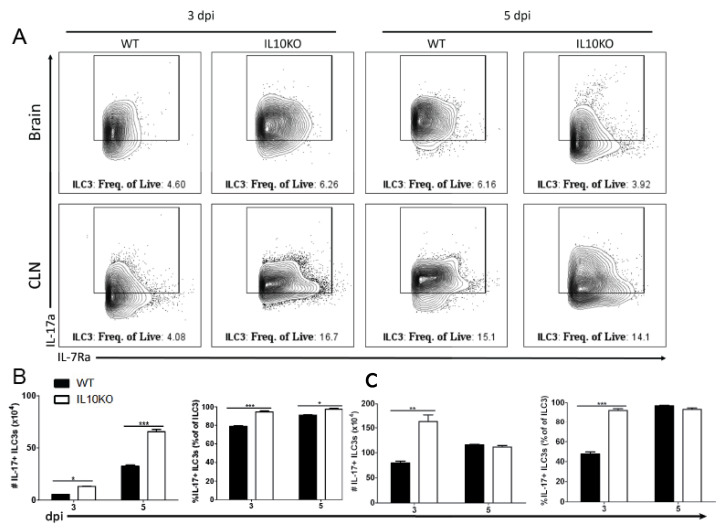
IL-17 production by type 3 innate lymphoid cells. Flow cytometric analysis of IL-17 production by ILC3s isolated and pooled from the brains and CLNs (n = 10) of WT (black bars) or IL10-deficient (white bars) mice 3 and 5 days after NSV infection. (**A**) Representative flow cytometry plots. Percentage and number of IL-17-expressing ILC3s isolated from brains (**B**) and CLNs (**C**). The data represent the mean ± SEM from three independent experiments. * *p* < 0.05, ** *p* < 0.01, *** *p* < 0.001.

*IL17a* mRNA is elevated in the brains of IL10-/- mice on day three after NSV infection preceding T cell recruitment to the CNS [22]. It was not determined which cell type was the early producer of IL-17A, but neutrophil depletion had no effect on disease outcome. Because ILC3s can produce IL-17A [49,50,51] and infiltrate the CNS early after SINV infection [25], we performed intracellular staining and flow cytometry on lymphocytes isolated from brains and CLNs (Figure 9A). On both days three and five after infection, there were higher percentages and numbers of ILC3s producing IL-17A in the brains of IL-10-deficient mice than WT mice (Figure 9B). In CLN, there were also elevated numbers and percentages of IL-17A-producing ILC3 cells in IL10-/- mice on day three but not day five after infection (Figure 9C). Therefore, ILC3s that are also more abundant in IL-10-deficient mice (Figure 3B) provide an early source of IL-17A in the CNS.

## 4. Discussion

**Figure 10:**
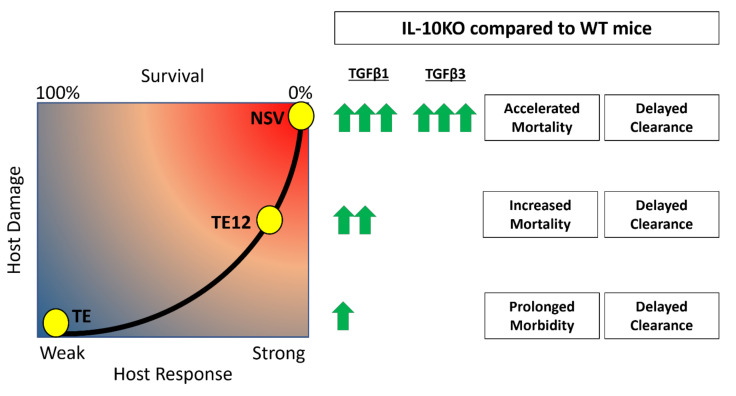
Accelerated mortality and delayed clearance associated with upregulation of TGFβ in the absence of IL-10. It is hypothesized that TGFβ1 and TGFβ3 are increased in the absence of IL-10 and lead to increased pathogenic Th17 responses, decreased antibody production, and more damage to the host. The concentrations of TGFb induced after infection are proportional to SINV strain virulence, with NSV being the most virulent and TE the least virulent.

A balance between inflammatory subsets of helper and regulatory T cells is crucial for survival from inflammatory diseases like viral encephalitis. In the center of this balance are IL-10 and TGFβ, two cytokines that regulate inflammation. Understanding how these two cytokines work in partnership during viral encephalomyelitis is key to understanding neuroprotection and clearance versus lethal immunopathologic disease. IL-10 is increased in the CNS during SINV encephalomyelitis and is an important modulator of CD4^+^ T cell responses and disease severity during CNS infection with strains of SINV that differ in virulence [22,25]. In the current study, we show that TGFβ1 and TGFβ3 are also upregulated in the CNS in response to SINV infection and that TGFβ1 is further increased in the absence of IL-10. Mice infected with NSV lacking IL-10 had increased production of TGFβ1 by ILC3s and T cells infiltrating the brain and spinal cord, decreased expression of surface molecules that prevent cleavage of pro-TGFβ on Tregs and increased activation of TGFβ downstream effector molecules. These changes were associated with increased expression of RORγt, *Pckα* mRNA and increased IL-17 production by ILC3s. Taken together, these results show induction and activation of TGFb in response to SINV infection of the CNS and a compensatory TGFb increase in the absence of IL-10 that may suppress inflammation but increase IL-17-related immunopathology and more rapidly promote fatal disease (Figure 10).

Previous studies of CNS viral infection in humans and mice have documented induction of TGFb expression with evidence of production by both intrinsic cells of the CNS as well as infiltrating inflammatory cells [52,53,54,55]. In CNS infection of mice with Theiler’s murine encephalomyelitis virus, neuronal production of TGFb is strain-dependent with the more virulent GDVII strain, but not the less virulent DA strain inducing TGFb expression and modulating inflammation [53]. In the current studies, neuronal production of TGFb1 was not assessed, but we documented production by infiltrating inflammatory cells associated with both innate and adaptive immune responses to infection. TGFb1-positive ILCs were detected 3 days after infection and were more abundant in the absence of IL-10. ILC3s are also important early sources of IL-17, and their dysregulation can lead to worse clinical outcomes [56]. Conversely, the lack of ILC3s inhibits the recruitment of helper T cells to the CNS during experimental autoimmune encephalomyelitis [49]. Infiltrating CD4^+^ and CD8^+^ T cells also produced TGFb with increases in the TGFb-producing CD4^+^ population associated with IL-10 deficiency.

TGFβ and IL-10 work in tandem to strike a balance between Th17 and regulatory T cells, and changes in this balance can determine the outcome of infection [21,57,58,59]. In vitro and in vivo studies have shown a dose-dependent effect of TGFb. Higher concentrations, in conjunction with other cytokines, lead to SMAD-independent induction of Th17 cell differentiation, whereas lower concentrations induce regulatory T cells through SMAD2/3 induction of FoxP3 [45,46,47,60,61]. For example, increased TGF-β leads to the development of pathogenic Th17 cells that are key to inducing autoimmunity [61]. The isoform of TGFβ could also impact Th17 cells as TGFβ3 is more efficient than TGFβ1 in the induction of pathogenic Th17 cells leading to autoimmunity [62].

In the absence of IL-10, it is postulated that TGFβ will compensate for the regulatory loss and perform similar but different functions that can lead to worse clinical outcomes [62]. In encephalomyelitis induced by the TE12 strain of SINV that has intermediate virulence, IL-10 deficiency led to more severe disease associated with an increase in Th1 responses [25]. In mice infected with the more virulent NSV, concentrations of TGFb1 increased in the absence of IL-10 with greater production of IL-17A and earlier appearance of more abundant pathogenic Th17 cells in the CNS [22]. Therefore, the effects of IL-10 deficiency on immunopathologic responses in the CNS during alphavirus infection are influenced by virus strain.

Understanding the context-dependent partnership between IL-10 and TGFβ will be important for the successful development of immunotherapies. These results have shown that there are consequences of eliminating a single cytokine, as another similar, yet diverging factor will be upregulated to take its place.

## Data Availability

Not applicable.

## Supplementary Materials

The following supporting information can be downloaded at: https://www.mdpi.com/article/10.3390/v14081791/s1.

## References

[1] Griffin D.E. (2011). Viral encephalomyelitis. PLoS Pathog..

[2] Griffin D.E. (2010). Emergence and re-emergence of viral diseases of the central nervous system. Prog. Neurobiol..

[3] Calisher C.H. (1994). Medically important arboviruses of the United States and Canada. Clin. Microbiol. Rev..

[4] Silverman M.A., Misasi J., Smole S., Feldman H.A., Cohen A.B., Santagata S., McManus M., Ahmed A.A. (2013). Eastern equine encephalitis in children, Massachusetts and New Hampshire, USA, 1970–2010. Emerg. Infect. Dis..

[5] Adams A.P., Navarro-Lopez R., Ramirez-Aguilar F.J., Lopez-Gonzalez I., Leal G., Flores-Mayorga J.M., da Rosa A.P.A.T., Saxton-Shaw K.D., Singh A.J., Borland E.M. (2012). Venezuelan equine encephalitis virus activity in the Gulf Coast region of Mexico, 2003–2010. PLoS Negl. Trop. Dis..

[6] Aguilar P.V., Estrada-Franco J.G., Navarro-Lopez R., Ferro C., Haddow A.D., Weaver S.C. (2011). Endemic Venezuelan equine encephalitis in the Americas: Hidden under the dengue umbrella. Future Virol..

[7] Adouchief S., Smura T., Sane J., Vapalahti O., Kurkela S. (2016). Sindbis virus as a human pathogen-epidemiology, clinical picture and pathogenesis. Rev. Med. Virol..

[8] Jackson A.C., Moench T.R., Trapp B.D., Griffin D.E. (1988). Basis of neurovirulence in Sindbis virus encephalomyelitis of mice. Lab. Investig. Tech. Methods Pathol..

[9] Jackson A.C., Moench T.R., Griffin D.E., Johnson R.T. (1987). The pathogenesis of spinal cord involvement in the encephalomyelitis of mice caused by neuroadapted Sindbis virus infection. Lab. Investig. Tech. Methods Pathol..

[10] Aguilar P.V., Greene I.P., Coffey L.L., Medina G., Moncayo A.C., Anishchenko M., Ludwig G.V., Turell M.J., O’Guinn M.L., Lee J. (2004). Endemic Venezuelan equine encephalitis in northern Peru. Emerg. Infect. Dis..

[11] Aguilar P.V., Weaver S., Turell M.J., O’Guinn M.L., Rios Z., Huaman A., Klein T.A., Tesh R.B., Watts D.M., Olson J. (2007). Endemic eastern equine encephalitis in the Amazon region of Peru. Am. J. Trop. Med. Hyg..

[12] Griffin D.E. (1976). Role of the immune response in age-dependent resistance of mice to encephalitis due to Sindbis virus. J. Infect. Dis..

[13] Tucker P.C., Strauss E.G., Kuhn R.J., Strauss J.H., Griffin D.E. (1993). Viral determinants of age-dependent virulence of Sindbis virus for mice. J. Virol..

[14] Lustig S., Jackson A.C., Hahn C.S., Griffin D.E., Strauss E.G., Strauss J.H. (1988). Molecular basis of Sindbis virus neurovirulence in mice. J. Virol..

[15] Sherman L.A., Griffin D.E. (1990). Pathogenesis of encephalitis induced in newborn mice by virulent and avirulent strains of Sindbis virus. J. Virol..

[16] Griffin D.E., Levine B., Tyor W.R., Tucker P.C., Hardwick J.M. (1994). Age-dependent susceptibility to fatal encephalitis: Alphavirus infection of neurons. Arch. Virol. Suppl..

[17] Kimura T., Griffin D.E. (2000). The role of CD8(+) T cells and major histocompatibility complex class I expression in the central nervous system of mice infected with neurovirulent Sindbis virus. J. Virol..

[18] Rowell J.F., Griffin D.E. (2002). Contribution of T cells to mortality in neurovirulent Sindbis virus encephalomyelitis. J. Neuroimmunol..

[19] Manivannan S., Baxter V.K., Schultz K.L., Slusher B.S., Griffin D.E. (2016). Protective effects of glutamine antagonist DON in mice with alphaviral encephalomyelitis. J. Virol..

[20] Greene I.P., Lee E.Y., Prow N., Ngwang B., Griffin D.E. (2008). Protection from fatal viral encephalomyelitis: AMPA receptor antagonists have a direct effect on the inflammatory response to infection. Proc. Natl. Acad. Sci. USA.

[21] Li M.O., Flavell R.A. (2008). Contextual regulation of inflammation: A duet by transforming growth factor-beta and interleukin-10. Immunity.

[22] Kulcsar K.A., Baxter V.K., Greene I.P., Griffin D.E. (2014). Interleukin 10 modulation of pathogenic Th17 cells during fatal alphavirus encephalomyelitis. Proc. Natl. Acad. Sci. USA.

[23] Kulcsar K.A., Griffin D.E. (2016). T cell-derived interleukin-10 is an important regulator of the Th17 response during lethal alphavirus encephalomyelitis. J. Neuroimmunol..

[24] Chaudhry A., Samstein R.M., Treuting P., Liang Y., Pils M.C., Heinrich J.-M., Jack R.S., Wunderlich F.T., Brüning J.C., Müller W. (2011). Interleukin-10 signaling in regulatory T cells is required for suppression of Th17 cell-mediated inflammation. Immunity.

[25] Martin N.M., Griffin D.E. (2018). Interleukin-10 Modulation of Virus Clearance and Disease in Mice with Alphaviral Encephalomyelitis. J. Virol..

[26] Couper K.N., Blount D.G., Riley E.M. (2008). IL-10: The master regulator of immunity to infection. J. Immunol..

[27] Donnelly R.P., Sheikh F., Kotenko S.V., Dickensheets H. (2004). The expanded family of class II cytokines that share the IL-10 receptor-2 (IL-10R2) chain. J. Leukoc. Biol..

[28] Zhou Z., Peng X., Insolera R., Fink D.J., Mata M. (2009). IL-10 promotes neuronal survival following spinal cord injury. Exp. Neurol..

[29] Morikawa M., Derynck R., Miyazono K. (2016). TGF-beta and the TGF-beta Family: Context-Dependent Roles in Cell and Tissue Physiology. Cold Spring Harb. Perspect Biol..

[30] Chen W., Ten Dijke P. (2016). Immunoregulation by members of the TGFbeta superfamily. Nat. Rev. Immunol..

[31] Li M.O., Wan Y.Y., Sanjabi S., Robertson A.K., Flavell R.A. (2006). Transforming growth factor-beta regulation of immune responses. Annu. Rev. Immunol..

[32] Wang R., Zhu J., Dong X., Shi M., Lu C., Springer T.A. (2012). GARP regulates the bioavailability and activation of TGFbeta. Mol. Biol. Cell.

[33] Yoshimura A., Wakabayashi Y., Mori T. (2010). Cellular and molecular basis for the regulation of inflammation by TGF-beta. J. Biochem..

[34] Lee P.W., Severin M.E., Lovett-Racke A.E. (2017). TGF-beta regulation of encephalitogenic and regulatory T cells in multiple sclerosis. Eur. J. Immunol..

[35] Wilbanks G.A., Streilein J.W. (1992). Fluids from immune privileged sites endow macrophages with the capacity to induce antigen-specific immune deviation via a mechanism involving transforming growth factor-beta. Eur. J. Immunol..

[36] Vitkovic L., Maeda S., Sternberg E. (2001). Anti-inflammatory cytokines: Expression and action in the brain. Neuroimmunomodulation.

[37] Tran D.Q., Andersson J., Wang R., Ramsey H., Unutmaz D., Shevach E.M. (2009). GARP (LRRC32) is essential for the surface expression of latent TGF-beta on platelets and activated FOXP3+ regulatory T cells. Proc. Natl. Acad. Sci. USA.

[38] Lee E.Y., Schultz K.L., Griffin D.E. (2013). Mice deficient in interferon-gamma or interferon-gamma receptor 1 have distinct inflammatory responses to acute viral encephalomyelitis. PLoS ONE.

[39] Thach D.C., Kimura T., Griffin D.E. (2000). Differences between C57BL/6 and BALB/cBy mice in mortality and virus replication after intranasal infection with neuroadapted Sindbis virus. J. Virol..

[40] Travis M.A., Sheppard D. (2014). TGF-beta activation and function in immunity. Annu. Rev. Immunol..

[41] Stockis J., Colau D., Coulie P.G., Lucas S. (2009). Membrane protein GARP is a receptor for latent TGF-beta on the surface of activated human Treg. Eur. J. Immunol..

[42] Sun L., Jin H., Li H. (2016). GARP: A surface molecule of regulatory T cells that is involved in the regulatory function and TGF-beta releasing. Oncotarget.

[43] Miller M.M., Petty C.S., Tompkins M.B., Fogle J.E. (2014). CD4+CD25+ T regulatory cells activated during feline immunodeficiency virus infection convert T helper cells into functional suppressors through a membrane-bound TGFbeta/GARP-mediated mechanism. Virol. J..

[44] Wang R., Kozhaya L., Mercer F., Khaitan A., Fujii H., Unutmaz D. (2009). Expression of GARP selectively identifies activated human FOXP3+ regulatory T cells. Proc. Natl. Acad. Sci. USA.

[45] Zhou L., Lopes J.E., Chong M.M., Ivanov I.I., Min R., Victora G.D., Shen Y., Du J., Rubtsov Y.P., Rudensky A.Y. (2008). TGF-beta-induced Foxp3 inhibits T(H)17 cell differentiation by antagonizing RORgammat function. Nature.

[46] Malhotra N., Kang J. (2013). SMAD regulatory networks construct a balanced immune system. Immunology.

[47] Ichiyama K., Sekiya T., Inoue N., Tamiya T., Kashiwagi I., Kimura A., Morita R., Muto G., Shichita T., Takahashi S. (2011). Transcription factor Smad-independent T helper 17 cell induction by transforming-growth factor-beta is mediated by suppression of eomesodermin. Immunity.

[48] Meisel M., Hermann-Kleiter N., Hinterleitner R., Gruber T., Wachowicz K., Pfeifhofer-Obermair C., Fresser F., Leitges M., Soldani C., Viola A. (2013). The kinase PKCalpha selectively upregulates interleukin-17A during Th17 cell immune responses. Immunity.

[49] Hatfield J.K., Brown M.A. (2015). Group 3 innate lymphoid cells accumulate and exhibit disease-induced activation in the meninges in EAE. Cell Immunol..

[50] Takatori H., Kanno Y., Watford W.T., Tato C.M., Weiss G., Ivanov I.I., Littman D.R., O’Shea J.J. (2009). Lymphoid tissue inducer-like cells are an innate source of IL-17 and IL-22. J. Exp. Med..

[51] Gladiator A., LeibundGut-Landmann S. (2013). Innate lymphoid cells: New players in IL-17-mediated antifungal immunity. PLoS Pathog..

[52] Rauch J., Steffen J.F., Muntau B., Gisbrecht J., Pörtner K., Herden C., Niller H.H., Bauswein M., Rubbenstroth D., Mehlhoop U. (2022). Human Borna disease virus 1 encephalitis shows marked pro-inflammatory biomarker and tissue immunoactivation during the course of disease. Emerg. Microbes Infect..

[53] Tsunoda I., Libbey J.E., Fujinami R.S. (2007). TGF-beta1 suppresses T cell infiltration and VP2 puff B mutation enhances apoptosis in acute polioencephalitis induced by Theiler’s virus. J. Neuroimmunol..

[54] Allen S.J., Mott K.R., Wechsler S.L., Flavell R.A., Town T., Ghiasi H. (2011). Adaptive and innate transforming growth factor beta signaling impact herpes simplex virus 1 latency and reactivation. J. Virol..

[55] Beckham J.D., Tuttle K., Tyler K.L. (2009). Reovirus activates transforming growth factor beta and bone morphogenetic protein signaling pathways in the central nervous system that contribute to neuronal survival following infection. J. Virol..

[56] Forkel M., Mjosberg J. (2016). Dysregulation of Group 3 Innate Lymphoid Cells in the Pathogenesis of Inflammatory Bowel Disease. Curr. Allergy Asthma Rep..

[57] McKinstry K.K., Strutt T.M., Buck A., Curtis J.D., Dibble J.P., Huston G., Tighe M., Hamada H., Sell S., Dutton R.W. (2009). IL-10 deficiency unleashes an influenza-specific Th17 response and enhances survival against high-dose challenge. J. Immunol..

[58] Korn T., Oukka M., Kuchroo V., Bettelli E. (2007). Th17 cells: Effector T cells with inflammatory properties. Semin. Immunol..

[59] Huber S., Gagliani N., Esplugues E., O’Connor W., Huber F.J., Chaudhry A., Kamanaka M., Kobayashi Y., Booth C.J., Rudensky A.Y. (2011). Th17 cells express interleukin-10 receptor and are controlled by Foxp3^−^ and Foxp3^+^ regulatory CD4^+^ T cells in an interleukin-10-dependent manner. Immunity.

[60] Yoshinaka Y., Takahashi Y., Nakamura S., Katoh I., Takio K., Ikawa Y. (1999). Induction of manganese-superoxide dismutase in MRC-5 cells persistently infected with an alphavirus, sindbis. Biochem. Biophys. Res. Commun..

[61] Lee Y., Awasthi A., Yosef N., Xiao S., Peters A., Wu C., Kleinewietfeld M., Kunder S., Hafler D.A., Sobel R.A. (2012). Induction and molecular signature of pathogenic TH17 cells. Nat. Immunol..

[62] Sharma M., Kaveri S.V., Bayry J. (2013). Th17 cells, pathogenic or not? TGF-beta3 imposes the embargo. Cell Mol. Immunol..

