# Supplementary figures and images for: Effect of IL-10 Deficiency on TGFβ Expression during Fatal Alphavirus Encephalomyelitis in C57Bl/6 Mice
Source: Viruses. 2022 Aug 16;14(8):1791. doi: 10.3390/v14081791 (PMC9416572; doi:10.3390/v14081791)

IL-10KO Mice, 5 dpi  
Example ILC-3 Gating Strategy

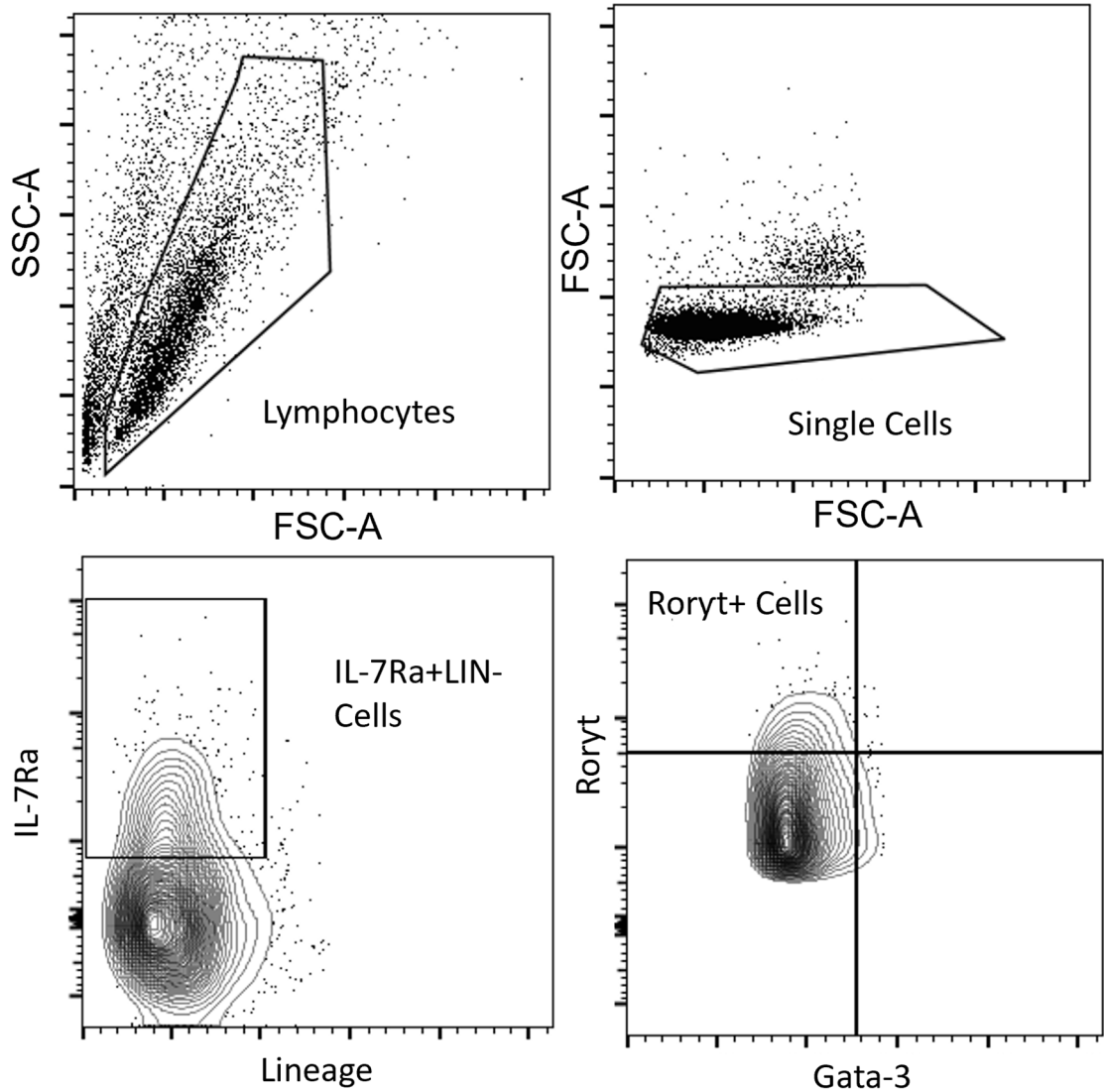

**Figure S1.** Example ILC3 gating strategy.

Supplement: Supplementary file 1 [file viruses-14-01791-s001.zip › viruses-1848422-supplementary.pdf]
